# Venous sinus stenting immediately reduces intracranial pressure in Idiopathic Intracranial Hypertension patients with venous sinus stenosis

**DOI:** 10.1186/2045-8118-12-S1-O62

**Published:** 2015-09-18

**Authors:** Samir A Matloob, Ahmed K Toma, Simon D Thompson, Chee L Gan, Edward W Dyson, Claudia Craven, Aswin Chari, Neekhil A Patel, Huan Wee Chan, Syed Shah, Patricia Haylock-Vize, Jinendra Ekanayake, Fergus Robertson, Lewis Thorne, Laurence D Watkins

**Affiliations:** 1 National Hospital for Neurology and Neurosurgery, UK

## Introduction

Idiopathic Intracranial Hypertension (IIH) is characterised by an increased intracranial pressure (ICP) in the absence of any central nervous system disease or structural abnormality, and normal CSF composition. Management becomes complicated once surgical intervention is required. Venous sinus stenosis has been suggested as a possible aetiology for IIH. Venous sinus stenting has emerged as a possible interventional option. Evidence for venous sinus stenting is based on elimination of the venous pressure gradient and clinical response. There have been no studies demonstrating the immediate effect of venous stenting on ICP.

## Methods

Patients with a potential or already known diagnosis of IIH were investigated according to departmental protocol. ICP monitoring was performed for 24 hours. When high pressures were confirmed, CT venogram and catheter venography were performed to look for venous stenosis to demonstrate a pressure gradient. If positive, venous stenting would be performed and ICP monitoring would continue for a further 24 hours after deployment of the venous stent.

## Results

Ten patients underwent venous sinus stenting with concomitant ICP monitoring. Nine out of ten patients displayed an immediate reduction in their ICP that was maintained at 24 hours. The average reduction in mean ICP and pulsatility was significant (p=0.003). Six out of ten patients reported a symptomatic improvement within the first 2 weeks.

## Conclusion

Venous sinus stenting results in an immediate reduction in ICP. This physiological response to venous stenting has not previously been reported. Venous stenting could offer an alternative treatment option in correctly selected patients with IIH.

